# Genetically Engineered Hypoimmune Human Muscle Progenitor Cells Can Reduce Immune Rejection

**DOI:** 10.1111/cpr.13802

**Published:** 2025-01-07

**Authors:** Yu Chen, Peng Wang, Shilin Ma, Chenran Yue, Xupeng Liu, Yeqian Cheng, Kun Liu, Tongbiao Zhao, Ng Shyh‐Chang

**Affiliations:** ^1^ Key Laboratory of Organ Regeneration and Reconstruction, State Key Laboratory of Stem Cell and Reproductive Biology, Institute of Zoology Chinese Academy of Sciences Beijing China; ^2^ Savaid Medical School University of Chinese Academy of Sciences Beijing China; ^3^ Beijing Institute for Stem Cell and Regenerative Medicine Beijing China

## Abstract

Cells face two challenges after transplantation: recognition and killing by lymphocytes, and cell apoptosis induced by the transplantation environment. Our hypoimmune cells aim to address these two challenges through editing of immunomodulatory proteins and overexpression of anti‐apoptotic proteins.

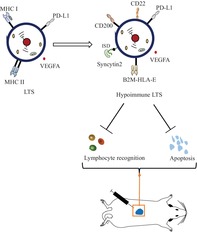

Dear Editor,

Muscle atrophy can lead to falls, accidental injuries, frailty, accelerated aging or even death in the elderly [1, 2, 3, 4, 5, 6, 7, 8, 9, 10, 11]. Others have demonstrated that single muscle stem cells can regenerate muscle fibres after transplantation, suggesting the feasibility of stem cell therapy [12, 13]. However, the loss of self‐renewal or differentiation potential of human muscle progenitor cells during in vitro expansion, and the immune rejection after in vivo transplantation, have limited the advancement of such therapies.

Our team previously rejuvenated human muscle stem cells through metabolic optimization using a minimal gene combination (LIN28A, TERT and sh‐P53: LTS), which can maintain self‐renewal and differentiation potential during long‐term in vitro culture [14], leaving immune rejection as the final unsolved problem. Therefore, we tried to obtain hypoimmune human muscle stem cells, based on LTS muscle cells. As shown in the schematic diagram (Figure 1a), we have identified a number of immunomodulatory proteins that need to be modified. By double knockout of B2M and CIITA, cells can avoid major histocompatibility complex (MHC)‐based recognition by T cells [15, 16]. However, this triggers NK cell‐mediated “missing self” killing responses [17, 18]. HLA‐E has been shown to bind to the inhibitory receptors CD94/NKG2A and LILRB1 (ILT2) on NK cells, to prevent NK cell killing [19, 20, 21]. Overexpressing a fusion of B2M joined to the extracellular domains of HLA‐E (B2M‐HLA‐E) reintroduces MHC class I expression without restoring all endogenous antigen presentation, after B2M knockout. PD‐L1 is an important immune checkpoint protein that can inhibit T cell activation and help tumour cells escape the immune system [22, 23, 24]. CD200 is an immunoregulatory “don't eat me” signal, that can inhibit the activation of macrophages and antigen presenting cells through the receptor CD200R [25, 26]. Syncytin2, expressed specifically in syncytiotrophoblasts during pregnancy, plays a vital role in preventing immune rejection of the placenta/foetus through its immunosuppressive domain (ISD) to resist T cell killing [27, 28, 29, 30, 31, 32]. CD22 participates in B cell inhibitory regulation [33, 34, 35]. After cell transplantation, particularly in xenotransplantation, multiple inflammatory cytokines could activate the NFkb pathway, leading to acute immune rejection and possibly transplanted cells' apoptosis [36, 37, 38]. Therefore, its negative regulator IKBa was also selected as a candidate gene. Transplanted cells may also die from a lack of nutrients, and thus VEGFA‐mediated vascularization and resolution of inflammation can help enhance the survival ability of transplanted cells [39, 40, 41, 42]. We planned to overexpress all of these proteins in LTS cells using piggyBac gene transfer technology, which provides support for large‐sized plasmid transgenesis [43, 44, 45]. Unexpectedly, we were surprised in an initial screen of immunoblotting antibodies, to find that LTS cells already endogenously upregulated PDL1, IkBa, and VEGFA, relative to normal muscle progenitors. These characteristics suggest that LTS cells may be ideal donors for engineering hypoimmune muscle progenitors (Figure S1). Hence, further studies prioritised the differential expression of CD22, HLA‐E, CD200, and Syncytin2.

**FIGURE 1:**
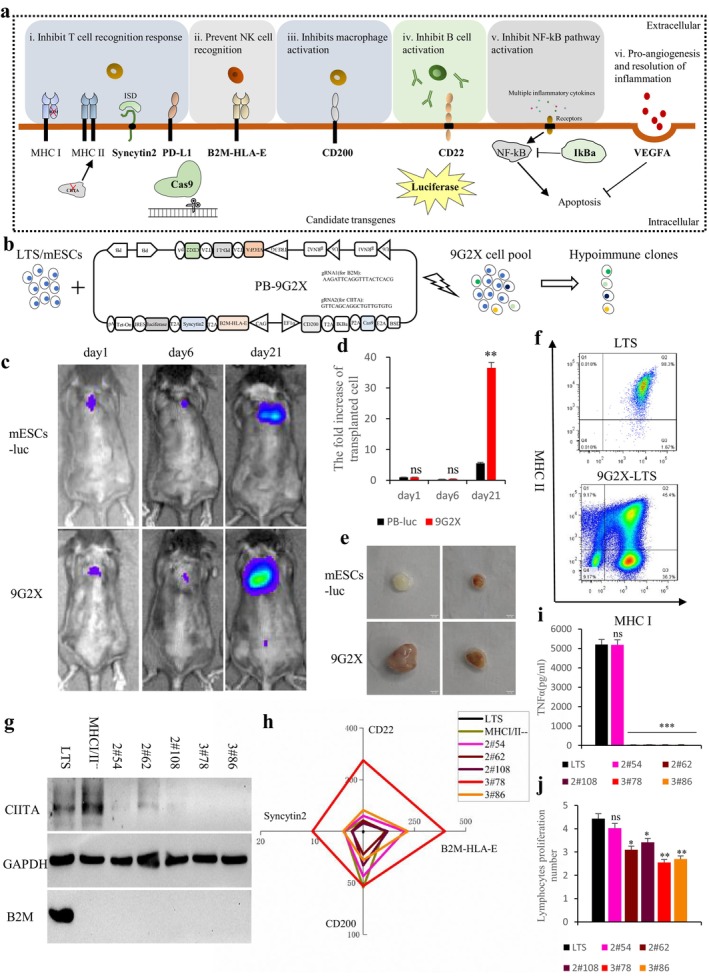
9G2X enhances the immune‐evasive abilities of mESCs/adult muscle progenitor. a. Candidate immunomodulatory factors and their role in innate and adaptive immune pathways. b. Schematic diagram of PB‐9G2X transposon plasmid construction, electroporation and hypoimmune cell clones screening. c. mESCs (luc vs. 9G2X) were transplanted subcutaneously into the back of allogeneic female C3H mice, and the survival of the transplanted cells in vivo was recorded by in vivo bioluminescence imaging (BLI). d. Statistical analysis of the data in (a) showed that the fluorescence signal of 9G2X mESCs could be maintained longer, relative to those co‐cultured with mESCs controls, *n* = 3, “ns” represents no significant difference, and “*” represents *p* < 0.05. e. Representative illustration of the remaining transplant grafts after dissection of allogeneic C3H mice on day 21. f. Identify MHCI‐MHCII‐ double negative cell populations and generate hypoimmune clones by flow cytometry. g. Western blot was used to verify the knockout of B2M and CIITA at the protein level in these hypoimmune cells. h. The expression levels of representative immunosuppressive factors in these clones were detected by qPCR and displayed as radar graphs. i. After mouse splenocytes were co‐cultured with LTS or hypoimmune cells for 5 days, the secretion level of TNFα in the culture supernatant was detected. The secretion of TNFα in groups 2#64, 2#108, 3#78 and 3#86 was significantly reduced, while that in group 2#54 was not significantly changed, relative to those co‐cultured with LTS controls, “ns” represents no significant difference, and “***” represents *p* < 0.001. j. Mouse splenocytes proliferation incubated with LTS or hypoimmune cells was measured by CFSE staining. Groups 2#64, 2#108, 3#78, and 3#86 reduced the potential for lymphocyte proliferation, while group 2#54 showed no significant change, relative to those co‐cultured with LTS controls, *n* = 3, “ns” represents no significant difference, “*” represents p < 0.05, and “**” represents *p* < 0.01. It is possible that clone 2#52 may have accumulated mutations that rendered its epitopes immunogenic.

Accordingly, we incorporated all candidate genes (Cas9 and luciferase were included as tools to facilitate gene knockout and transplant visualisation, respectively) and gRNAs into the piggyBac transposon plasmid, and obtained 9G2X‐LTS cells after electroporation and drug‐resistance selection. Next, we obtained multiple hypoimmune 9G2X‐LTS cell clones through FACS screening. Immunosuppressive factors were confirmed to effectively reduce the immunogenicity of human muscle progenitor cells through in vitro immune response testing with activated lymphocytes. Additionally, in vivo experiments confirmed that reducing the immune‐mediated apoptosis response can effectively improve the survival potential and growth of transplanted muscle cells. Overall, this study validates the feasibility of constructing hypoimmune muscle progenitor cells and provides valuable experimental data for future clinical applications.

According to previously published protocols [46, 47], we designed and synthesised the piggyBac transposon plasmid with all the nine transgenes and two gRNAs we needed, PB‐9G2X. To verify the immune‐evasive abilities of 9G2X after cell transplantation, we attempted to perform transplantation assays using mESCs in allogeneic mice. We allogeneically transplanted 1 million C57BL/6 mESCs (Luc vs. 9G2X) into female 129 and C3H mice, and detected cell survival or growth by in vivo bioluminescence imaging (BLI). The results showed that the 9G2X group had stronger survival potential and formed larger teratomas (Figure 1c–e, S2). This result encouraged us to develop hypoimmune muscle progenitor cells through 9G2X. We obtained 9G2X‐LTS cells through blasticidin drug screening post‐electroporation (Figure 1b, S3a). All 9 transgenes were overexpressed, and the cells retained normal differentiation potential (Figure S3b–c). To obtain single cell clones with confirmed double knockout of B2M (MHC I) and CIITA (MHC II), we performed fluorescence‐activated cell sorting (FACS) on IFN‐γ pre‐treated 9G2X‐LTS cells with MHCI/II antibodies, and isolated MHCI‐ MHCII‐ single cells (Figure 1f). Subsequently, after expansion of the single cells, we performed B2M and CIITA target gene sequencing (Figure S4) and protein expression validation by immunoblotting of the clonal lines (Figure 1g). All examined cell‐lines had stable knockout of B2M and CIITA by CRISPR‐Cas9. As mentioned above, since PDL1, IkBa and VEGFA were already highly expressed in LTS, thus we focused on comparing the expression levels of the other four transgenes in candidate hypoimmune cells. Quantitative RT‐PCR further confirmed the overexpression of those transgenes, with 3#78 showing the highest expression level of transgenes (Figure 1h).

Given ethical and regulatory constraints, human peripheral blood lymphocytes did not suffice for our experimental needs with regards to numbers, consistency and immune cell diversity. To mimic the complex immune challenges post‐transplantation, we assessed the immunogenicity of 9G2X cells through co‐culture with mouse spleen lymphocytes in vitro. The results showed that, except for clone 2#54, the supernatant TNFα secretion after co‐culture of the splenocytes with hypoimmune cells (clone 2#62, 2#108, 3#78 and 3#86) was significantly reduced (Figure 1i). Next, we measured the proliferation potential of mouse lymphocytes by CFSE staining. Consistent with the TNFα results, all the hypoimmune cells except clone 2#54 significantly inhibited the proliferation of splenocytes (Figure 1j). Thus, 4/5 of our candidate hypoimmune cells passed the in vitro testing.

It was previously shown that clonal cells with the highest levels of transgene expression also tend to have the strongest immunosuppressive potential [48]. Therefore, we prioritised hypoimmune clone 3#78 with the highest overall transgene expression for further verification. First, we tested the ability of clone 3#78 to resist killing by mouse splenocytes. The results showed that clone 3#78 showed stronger proliferation than LTS controls under co‐culture conditions with lymphocytes, indicating that hypoimmune cells can resist immune cytotoxicity (Figure 2a). We then evaluated the response of co‐cultured mouse splenocytes. CD107a is a hallmark marker of lymphocytes degranulation, reflecting the level of lymphocytes activation. The percentage of CD107a + lymphocytes co‐cultured with clone 3#78 decreased significantly relative to those co‐cultured with LTS controls, indicating that lymphocyte activation was inhibited by the 9G2X cassette (Figure 2b). In addition, the level of annexin‐V+ lymphocytes was significantly increased during co‐culture with clone 3#78, relative to LTS controls (Figure 2c). We also measured changes in the density of lymphocytes per unit area under co‐culture conditions to infer cell survival. The results showed that the number of cells per unit area of lymphocytes in the clone 3#78 group was significantly reduced compared with the LTS controls, indicating that hypoimmune 9G2X cells can inhibit lymphocyte survival (Figure 2d). In short, generation of hypoimmune adult muscle progenitor cells significantly reduced immune responses in vitro. Next, to assess the survival potential of cells after in vivo transplantation, we used the LTS control cell line for transplantation experiments. Given the above findings, we anticipate that these hypoimmune human muscle progenitor cells will evade rejection following transplantation into mice with intact immune systems. Regrettably, the cells were rapidly rejected following transplantation into wildtype C57BL/6 mice within 2 days, preventing any quantitative analysis.

**FIGURE 2:**
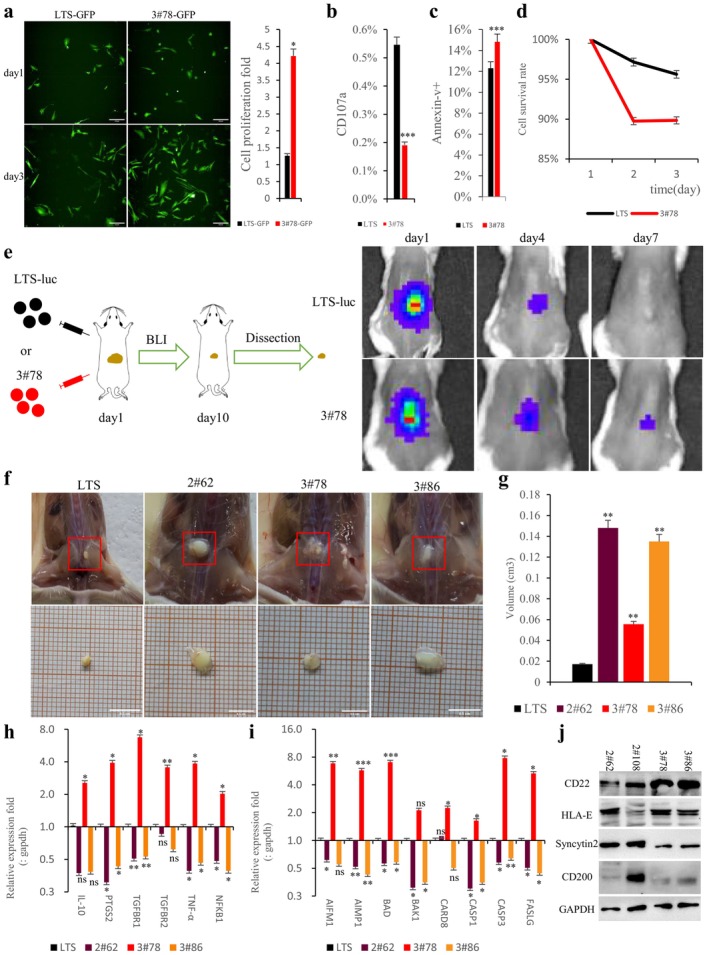
Hypoimmune muscle progenitors have stronger survival potential in vivo. a. Representative images of LTS‐GFP or hypoimmune clone 3#78‐GFP co‐cultured with mouse splenocytes. Statistical analysis of the corresponding proliferation capacity of target cells, *n* = 3, *p* < 0.05. b. Detection of CD107a positive rate of mouse splenocytes co‐cultured with LTS or hypoimmune clone 3#78, *n* = 3, *p* < 0.001. c. After mouse splenocytes were co‐cultured with LTS or hypoimmune clone 3#78 for 48 h. Annexin‐v positive rate of lymphocytes was detected, *n* = 3, *p* < 0.001. d. Changes in the number of cells per unit area during co‐culture of mouse splenocytes with LTS or hypoimmune clone 3#78, *n* = 3, *p* < 0.05. e. LTS‐luc and hypoimmune clone 3#78 in vivo validation design and representative images of BLI. f. Representative diagram of mouse dissection and sampling 11 days after LTS and hypoimmune cell transplantation. g. Determination of LTS and hypoimmune cell graft volume. The hypoimmune cell graft volume was significantly larger than that of the LTS control group, *n* = 3, *p* < 0.01. In addition, the residual graft volume of 2#62 and 3#86 is also significantly larger than that of 3#78 (not shown in the figure, 2#62 vs. 3#78 (*p* < 0.01), 3#86 vs. 3#78 (*p* < 0.05)). h. Detection of inflammation levels in graft. The inflammation levels of hypoimmune clones 2#62 and 3#86 were significantly reduced, *n* = 3, “ns” represents no significant difference, “*” represents *p* < 0.05, “**” represents *p* < 0.01, and “***” represents *p* < 0.001. i. Pro‐apoptotic factor expression detection, and they showed a significantly down‐regulation trend in 2#62 and 3#86, *n* = 3, “ns” represents no significant difference, “*” represents *p* < 0.05, “**” represents *p* < 0.01, and “***” represents *p* < 0.001. j. Detection of protein expression levels of immunosuppressive factors in hypoimmune cells.

Interestingly, we discovered that the remaining immune cells in NOD/Shi‐scid IL2Rγ^null^ (NOG) mice, including some NK, T and B cells, could only mount a slower adaptive response against human LTS cells, leading to their immune rejection over 7 days. This finding suggests that NOG mice can serve as a model of immunosuppression to test LTS and 9G2X‐LTS cell transplantation. First, we transplanted 5 million cells into the subcutaneous tissue of the back of female NOG mice and detected cell survival by BLI on days 1, 4, and 7. The hypoimmune clone 3#78 showed stronger survival ability than LTS controls (Figure 2e).

Encouraged by these results, we wanted to know whether other hypoimmune clones could have better survival potential after transplantation as well. We transplanted hypoimmune clones 2#62, 2#108, 3#78 and 3#86 into female NOG mice. On the 11th day after transplantation, the specimens were dissected and the volume of residual cell grafts was measured using water displacement (Figure 2f). The results showed that the volume of residual cell grafts of hypoimmune clones 2#62, 3#78 and 3#86 was significantly larger than that of LTS controls (*p* < 0.05) (Figure 2f,g). Next, we detected the expression differences of inflammation and apoptosis genes in the grafts, and the results showed that the inflammation levels of 2#62 and 3#86 were significantly lower than 3#78 (Figure 2h). In parallel, the pro‐apoptotic genes of 2#62 and 3#86 were also significantly lower than 3#78, suggesting that the larger grafts were due to lower rates of immunity‐induced apoptosis after transplantation (Figure 2i). Surprisingly, clone 2#108 did not survive post‐transplantation in vivo. To elucidate the reasons behind the variance in graft volumes across different hypoimmune clones, we ascertained the actual protein expression levels of these transgenes using immunoblots. Our findings revealed that clone 2#62 exhibited superior Syncytin2 protein expression compared to clone 3#78. Concurrently, clone 3#86 demonstrated enhanced expression of CD22 and CD200 proteins over clone 3#78. For clone 2#108, their low levels of HLA‐E may have triggered their “missing self” rejection by NK cells after transplantation, indicating that HLA‐E is necessary to resist xenogeneic rejection (Figure 2j).

In summary, this study showed that genetic engineering of human muscle progenitor cells, by knocking out B2M and CIITA and overexpressing immunomodulatory transgenes (B2M‐HLA‐E, CD200, Syncytin2 and CD22) can substantially decrease their immunogenicity. This modification also dampens the immune response when these cells are co‐cultured with mouse splenocytes in vitro and improves their survival rates following in vivo transplantation (Figure S5). In addition, we found that random differences in the expression of different immunomodulatory proteins also affect the growth/survival rate of cells after transplantation by changing the degree of apoptosis after cell transplantation. Of course, we still face unresolved issues, such as the lack of a humanised mouse model to accurately assess cell transplantation survival and the absence of optimal culture conditions for macrophages and B cells, which hindered precise testing of CD22 and CD200 effects. Based on the phenotypes in NOG mice, we did not pursue mild busulfan treatment, low‐dose irradiation, or other milder immunosuppression models with stronger immune systems that might obscure the benefits of 9G2X. These challenges will be priorities in our future research. In summary, our work offers valuable insights for obtaining hypoimmune human progenitor cells for transplantation in vivo in the future.

## Author Contributions

Y.C. designed and performed the experiments, and drafted the manuscript. P.W. and T.Z. assisted in completing the manuscript. P.W., S.M., C.Y., X.L., Y.C. and K.L. helped with the experiments. N.S.C. designed and supervised the entire project.

## Conflicts of Interest

Ng Shyh‐Chang is an Editorial Board member of Cell Proliferation and a co‐author of this article. He was excluded from the editorial decision‐making related to the acceptance of this article for publication in the journal. All other authors declare no conflicts of interest.

## Supporting information


**Data S1** Supporting Information.

**Data S2** Supporting figures.

## Data Availability

The data that support the findings of this study are available from the corresponding author upon reasonable request.
