# Supplementary material for: Genetically Engineered Hypoimmune Human Muscle Progenitor Cells Can Reduce Immune Rejection
Source: Cell Prolif. 2025 Jan 7;58(4):e13802. doi: 10.1111/cpr.13802 (PMC11969239; doi:10.1111/cpr.13802)
Supplement: Supplementary file 1 — Data S1 Supporting Information. [file CPR-58-e13802-s001.docx]

**Supplementary Material**

**MATERIALS AND METHODS**

**Cell culture and differentiation**

The human muscle progenitor cells used in this article were derived from young adult primary HSKM progenitor cells isolated from the quadriceps muscle of a 20-year-old female subject and forced to express the minimal transgene combination required for rejuvenation (LIN28A, TERT, and sh-P53 (LTS)) ^1^. The experimental procedures meet the standards of the General Requirements for Stem Cells^2^. They were incubated at 37°C, 5% CO2 in a growth medium consisting of Dulbecco's modified Eagle's medium (DMEM)/F-12 (Gibco) containing 20% ​​fetal bovine serum (FBS; GE Healthcare), 1% L-glutamine (Gibco), and 1% penicillin-streptomycin (Gibco). At each passage, when the cells reached 80% confluence, they were digested with 0.25% trypsin (Gibco) and diluted 1:10. When cells reached 80%–100% confluence, differentiation was initiated by replacing the growth medium with differentiation medium consisting of DMEM/F-12, 2% KnockOut Serum Replacement (Gibco), 1% L-glutamine (Gibco), and 1% penicillin-streptomycin (Gibco).Mouse embryonic stem cells were generously supplied by TB Zhao's lab and cultured in accordance to Requirements for Stem Cells^3^.

**Mice**

All female mice used in this article were 2-3 months old and were ordered from Vital River. Mice were housed in a specific pathogen-free animal facility. All experiments were performed in accordance with protocols approved by the Animal Care and Use Committee of the Institute of Zoology, Chinese Academy of Sciences.

**Hypoimmune PiggyBac transposon plasmid design and construction**

All transgenic CDS sequence information was obtained from NCBI (see supplementary information) and synthesized by BGI. Every 3-4 transgenes were combined into an open reading frame region, which was driven by different promoters. Based on the design of Liu^4^ , the transgenes in each open reading frame region were separated by "self-cleaving" 2A peptides (P2A, E2A, and T2A) or IRES. The gRNA sequence of B2M or CIITA was designed on CRISPOR (http://crispor.tefor.net/crispor.py) and driven by the U6 promoter. The above sequences were placed in the PiggyBac transposon plasmid (System Biosciences) to complete the plasmid construction.

**Transgenic cell line construction**

We extracted high-concentration 9G2X plasmid (MACHEREY NAGEL), took 30ug, and completed electroporation using the Lonza 2b nucleofector, V-013 program (electroporation reagent, VPI-1002). After 48h, the 9G2X-LTS cell line was obtained by blasticidin drug selection. GFP virus packaging was completed by lentiviral plasmid (Addgene No. 19119), dR8.2 packaging plasmid (Addgene No. 8455), VSV-G envelope plasmid (Addgene No. 8454) and pLenti CMV GFP Blast (659-1; Addgene No. 17445). The viral supernatant was collected within the 48-hour to 96-hour window and filtered with a 0.45 μm filter (Sartorius). Three days after viral transduction of cells, GFP-positive cells were sorted by flow cytometry for use.

**Flow cytometry**

After four days of treatment with Interferon-γ (peprotech, 50 ng/ml), the 9G2X-LTS cell line was digested with 0.25% trypsin (Gibco) and dispersed into single cells. The cells were incubated with fluorescent conjugated antibodies FITC HLA-ABC (Invitrogen, 11-9983-42) and PE HLA-DR (Biolegend, 327007) for 30 minutes at 4°C. The harvested cells were sorted by flow cytometry for MHCI&MHCII-double negative cells using a BD LSR Fortessa x-20 analyzer, and single cells were seeded in 96-well plates (coring).

**Gene knockout detection**

The single cells to be inoculated in 96-well plates were grown into clones, and the cell clones were digested. Half of the cells were kept as seeds, and the other half were lysed by proteinase K lysis buffer to obtain genomic PCR. Specific verification primers were designed based on the sequence bound by gRNA on the B2M/CIITA genome (B2M F: AAGCTGACAGCATTCGGGC, R: AACCACAACCATGCCTTACTTT; CIITA F: CATCCTTGGGGAAGCTGAGG, R: ATGTTTGCTCGGGAGGTCAG). Using the verification primers, PCR reaction was performed on the genomic DNA of the hypoimmune clones. Then, the PCR products were sequenced by the second generation, and the sequences were compared and analyzed using Vector NTI software.

**Western blot**

After 4 days of IFN-γ treatment, proteins were extracted using RIPA buffer (Thermo) supplemented with protease inhibitors (Thermo) and phosphatase inhibitors (Thermo). Proteins were quantified using the Pierce BCA protein assay kit (Thermo) and analyzed using a Sunrise Tecan plate reader. After SDS-PAGE gel electrophoresis and electrotransfer to nitrocellulose membranes (GE Healthcare), Western blots were performed using the following primary antibodies and dilutions: CIITA (CST, 3739, 1:1000), B2M (Abcam, ab75853, 1:1000), CD22 (Abcam, ab20772,1:1000), CD200 (Abcam, ab254193,1:1000), IKBa (CST, 4812, 1:1000), Syncytin2 (Abcam, ab230235,1:1000), HLA-E (Santa cruz, sc-71262, 1:1000), GAPDH (CST, 5174, 1:000). Blots were stained with horseradish peroxidase-conjugated secondary antibodies (CST, 7074P2, 1:1000) and visualized using ECL ultraluminescent solution (LABLEAD) on a FL1000 imaging system (Thermo).

**Quantitative PCR**

RNA was extracted with TRIzol (Thermo), and genomic DNA removal and reverse transcription were performed according to the manufacturer's instructions (Takara, RR047B). The resulting cDNA was diluted 10 times and then qPCR was performed using SYBR Green Master (Yeasen) on a real-time fluorescence quantitative PCR instrument (LC 480Ⅱ, Roche) according to the manufacturer's instructions.

**Immune cell extraction and activation**

All mice were purchased from Vital River. They were fully anesthetized with isopentane and then quickly killed by dislocation. After spraying alcohol thoroughly, the spleen was dissected and removed using pre-autoclaved surgical scissors and forceps. The spleen was washed repeatedly 5 times in PBS supplemented with 5% penicillin-streptomycin and placed in a sterile and clean 10cm dish. The spleen was repeatedly ground using the rubber stopper of a 10ml syringe until there were no large particles. A 50ml centrifuge tube was taken, and a 70um cell strainer was placed on it. The grinding solution was transferred to the strainer to filter and obtain single cells. Red blood cells were lysed using red blood cell lysis buffer according to the instructions (Solarbio). Then, T cells and NK cells were sorted by magnetic beads (PBM, 720305, 720705). RPMI 1640 medium (Gibco) was used, supplemented with 10% fetal bovine serum (FBS; GE Healthcare) and 1% penicillin-streptomycin (Gibco). T cells were inoculated in anti-CD3/CD28 pre-coated culture dishes^5^.

**Cell killing assay**

Target cells were pre-seeded in 96-well plates at 500 cells/well. After overnight adherence, immune cells were added at a ratio of 1:10 for co-culture. The proliferation of target cells was recorded using a dual-disc confocal laser high-content imaging analysis system (PE, Opera Phenix).

**Immune cell status detection**

After 48 hours, the co-cultured immune cells were sampled and stained with FITC-CD107a (Biolegend, 121605) or FITC-Annexin-v/PI (Yeasen) and analyzed by flow cytometry. After 72 hours, the supernatant was collected and the TNFα concentration was determined according to the instructions (Cloud Clone). For immune cell proliferation assays, the change in the number of cells per unit area was recorded by a CCD camera (Nikon) and statistically analyzed in ImageJ. CFSE (Thermo) was pre-stained, and the fluorescence intensity was statistically analyzed by flow cytometry on day 1/day 3, and analyzed using Flowjo.

**Cell transplantation and in vivo imaging**

Quantitatively 5 x10^5^ cells to be transplanted were resuspended in a matrigel-PBS mixture (Stemcell, 1:1) and transplanted flatly into the back subcutaneous tissue of NOG mice (ordered from Vital River). The images were recorded and quantitatively analyzed using a small animal in vivo imaging system (IVIS Spectrum) on day 1, day 4, and day 7.

**Tissue sampling and processing**

TRIzol (Thermo) and zirconium oxide beads (AORAN, 3 mm) were added to the collected tissues, and the tissue samples were homogenized by a tissue grinder, and RNA was extracted and qPCR procedures were performed according to the above protocol.

**References**

1. Wang, P., Liu, X., Chen, Y., Jun-Hao, E.T., Yao, Z., Min-Wen, J.C., Yan-Jiang, B.C., Ma, S., Ma, W., Luo, L., et al. (2023). Adult progenitor rejuvenation with embryonic factors. Cell Prolif *56*, e13459. 10.1111/cpr.13459.

2. Hao, J., Ma, A., Wang, L., Cao, J., Chen, S., Wang, L., Fu, B., Zhou, J., Pei, X., Zhang, Y., et al. (2020). General requirements for stem cells. Cell Prolif *53*, e12926. 10.1111/cpr.12926.

3. Hao, J., Cao, J., Wang, L., Ma, A., Chen, S., Ding, J., Wang, L., Fu, B., Zhang, Y., Pei, X., et al. (2020). Requirements for human embryonic stem cells. Cell Prolif *53*, e12925. 10.1111/cpr.12925.

4. Liu, Z., Chen, O., Wall, J.B.J., Zheng, M., Zhou, Y., Wang, L., Vaseghi, H.R., Qian, L., and Liu, J. (2017). Systematic comparison of 2A peptides for cloning multi-genes in a polycistronic vector. Sci Rep *7*, 2193. 10.1038/s41598-017-02460-2.

5. Nan, X., Zhang, B., Hao, J., Yue, W., Fu, B., Qu, M., Zhang, Y., Wang, H., Fang, F., Wei, J., et al. (2022). Requirements for human haematopoietic stem/progenitor cells. Cell Prolif *55*, e13152. 10.1111/cpr.13152.
