# Supplementary material for: Genetically Engineered Hypoimmune Human Muscle Progenitor Cells Can Reduce Immune Rejection
Source: Cell Prolif. 2025 Jan 7;58(4):e13802. doi: 10.1111/cpr.13802 (PMC11969239; doi:10.1111/cpr.13802)
Supplement: Supplementary file 2 — Data S2 Supporting figures. [file CPR-58-e13802-s002.pdf]

S1. LTS cells are ideal donors for engineering hypoimmune muscle progenitor cells.

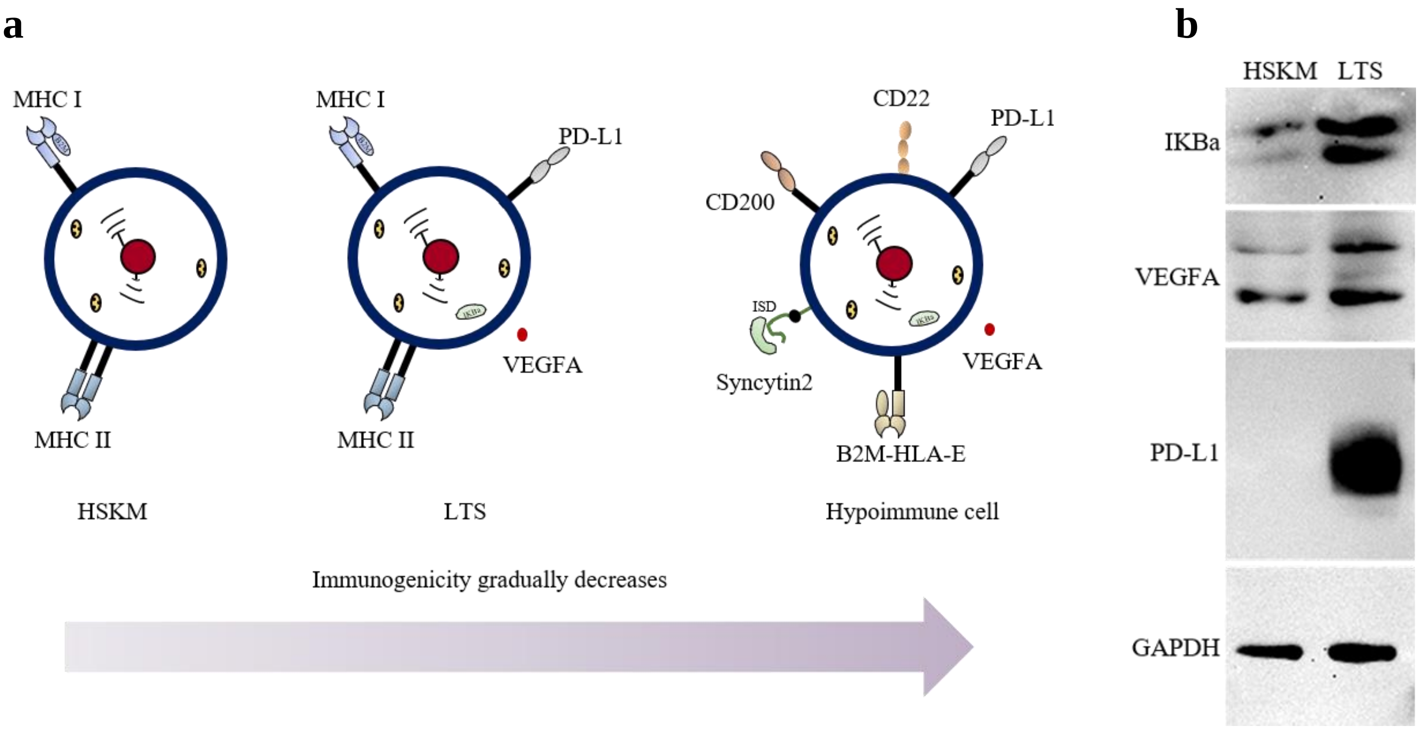

FigS1.

a. Based on the expression levels of immunosuppressive proteins, we speculate that the immunogenicity of HSKM vs LTS vs Hypoimmune cells gradually decreases. **b.** The immunosuppressive factors IKBa, VEGFA, and PD-L1 are upregulated in LTS cells.

S2. 9G2X can enhance the survival potential of mESCs after allogeneic transplantation

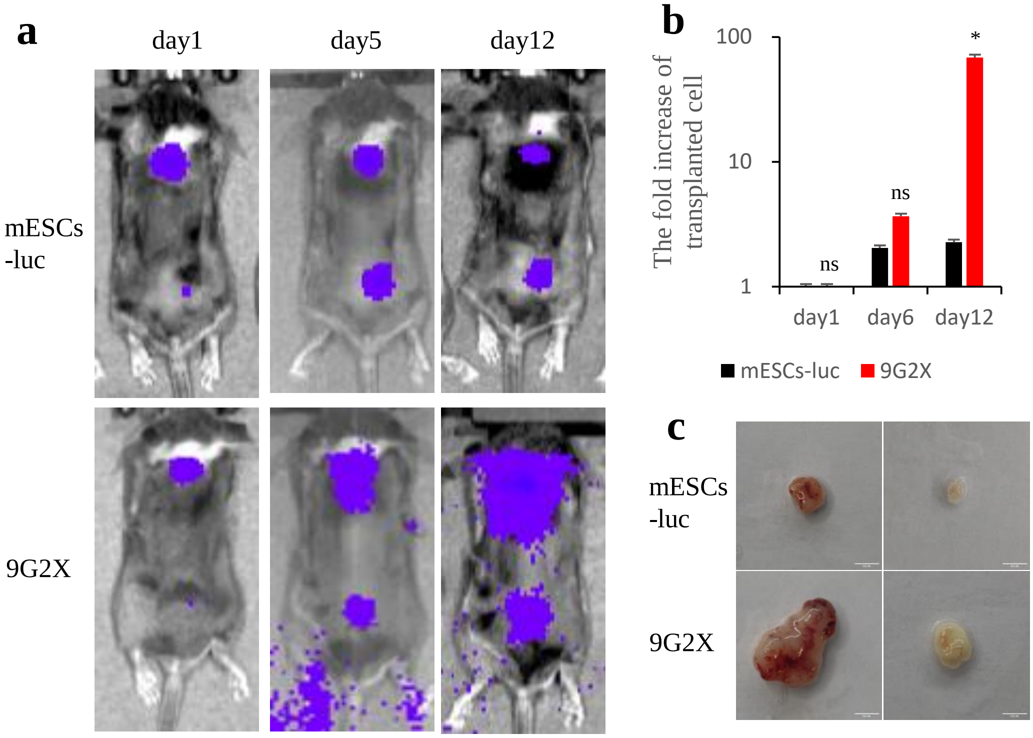

FigS2.

a. mESCs (luc vs 9G2X) were transplanted subcutaneously into the back of allogeneic female 129 mice, and the survival of the transplanted cells in vivo was recorded by BLI.

b. Statistical analysis of the data in (a) showed that the fluorescence signal of 9G2X mESCs could be maintained longer, n=3, "ns" represents no significant difference, and "**" represents $p<0.01$.

c. Representative illustration of the remaining transplant grafts after dissection of allogeneic 129 mice on day 21.

S3. Verification of transgenic expression and differentiation potential of 9G2X-LTS cells

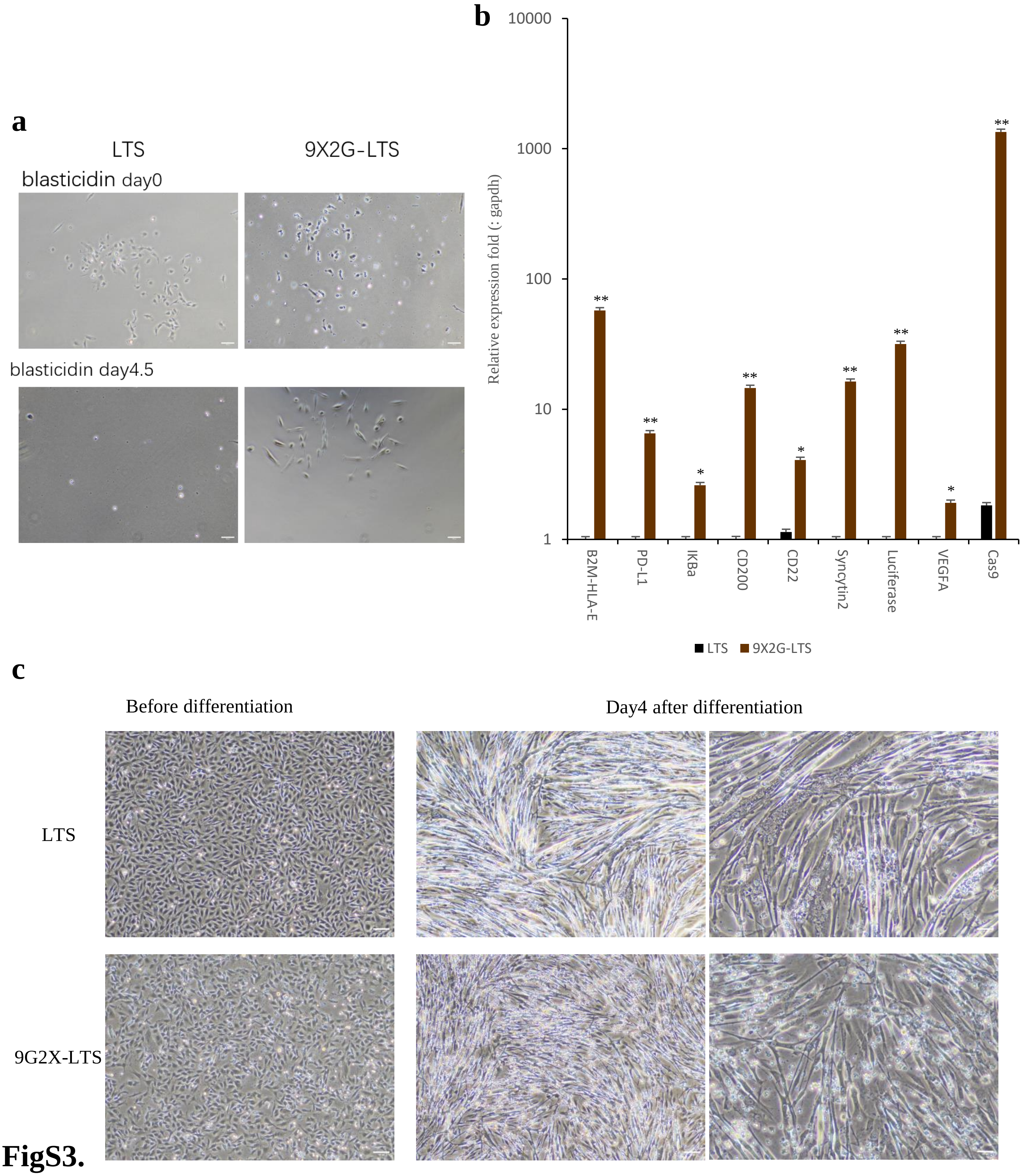

FigS3.

a. Schematic diagram of 9G2X-LTS cell drug screening.

b. The transgenes in the 9G2X-LTS cell line were successfully overexpressed

c. 9G2X-LTS cell line can differentiate normally.

S4. PCR detection of gene knockout in hypoimmune cells

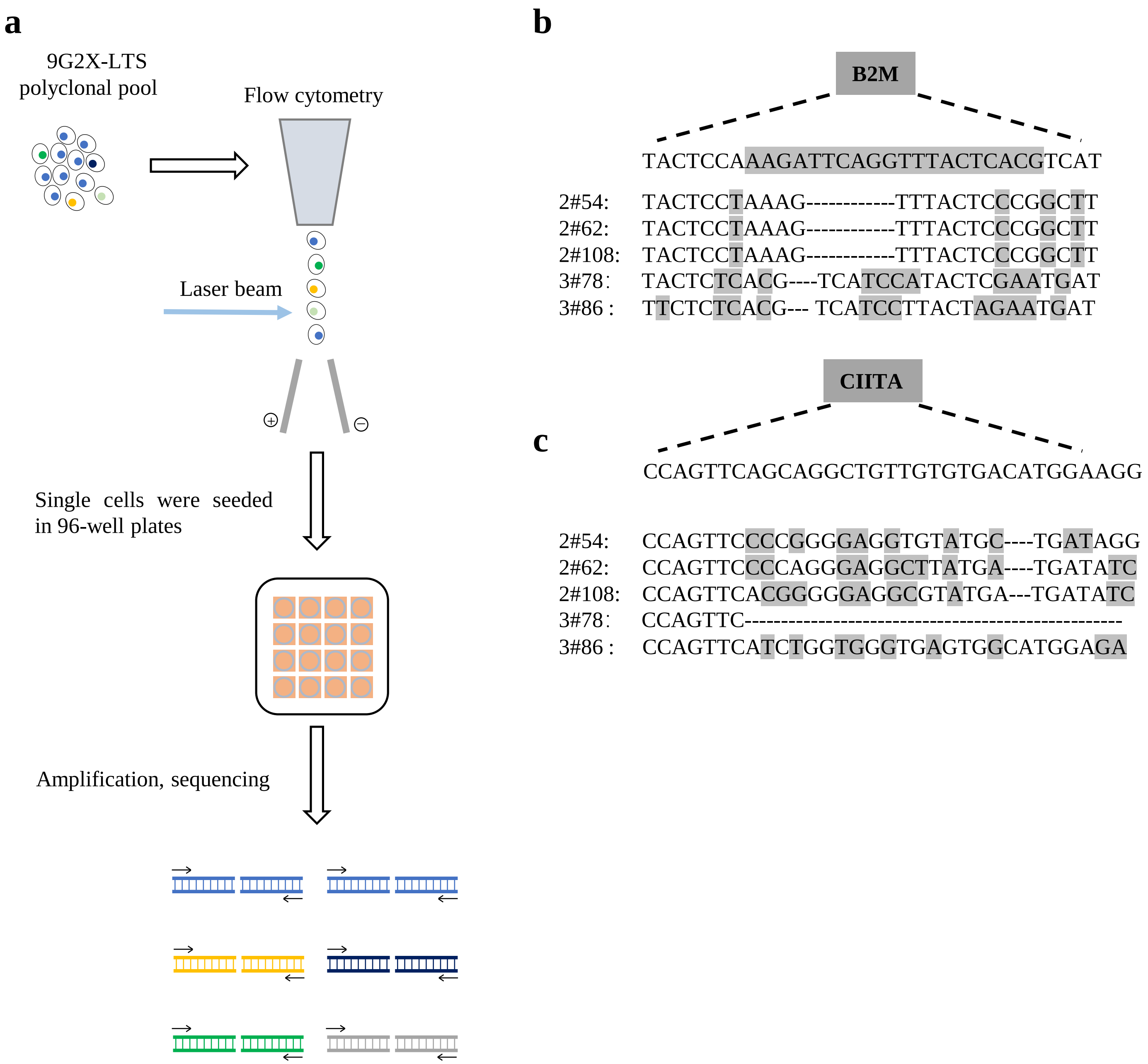

FigS4.

a. Schematic diagram of obtaining hypoimmune cell clones. CD22+MHCI/II- cells were sorted by flow cytometry, seeded in 96-well plates, and single cell clones were obtained and verified by sequencing.

b-c. Genomic DNA was extracted after monoclonal cell amplification, and the products amplified by PCR were sequenced for verification.

S5. Schematic diagram of hypoimmune muscle progenitor cells reducing xenogeneic immune rejection

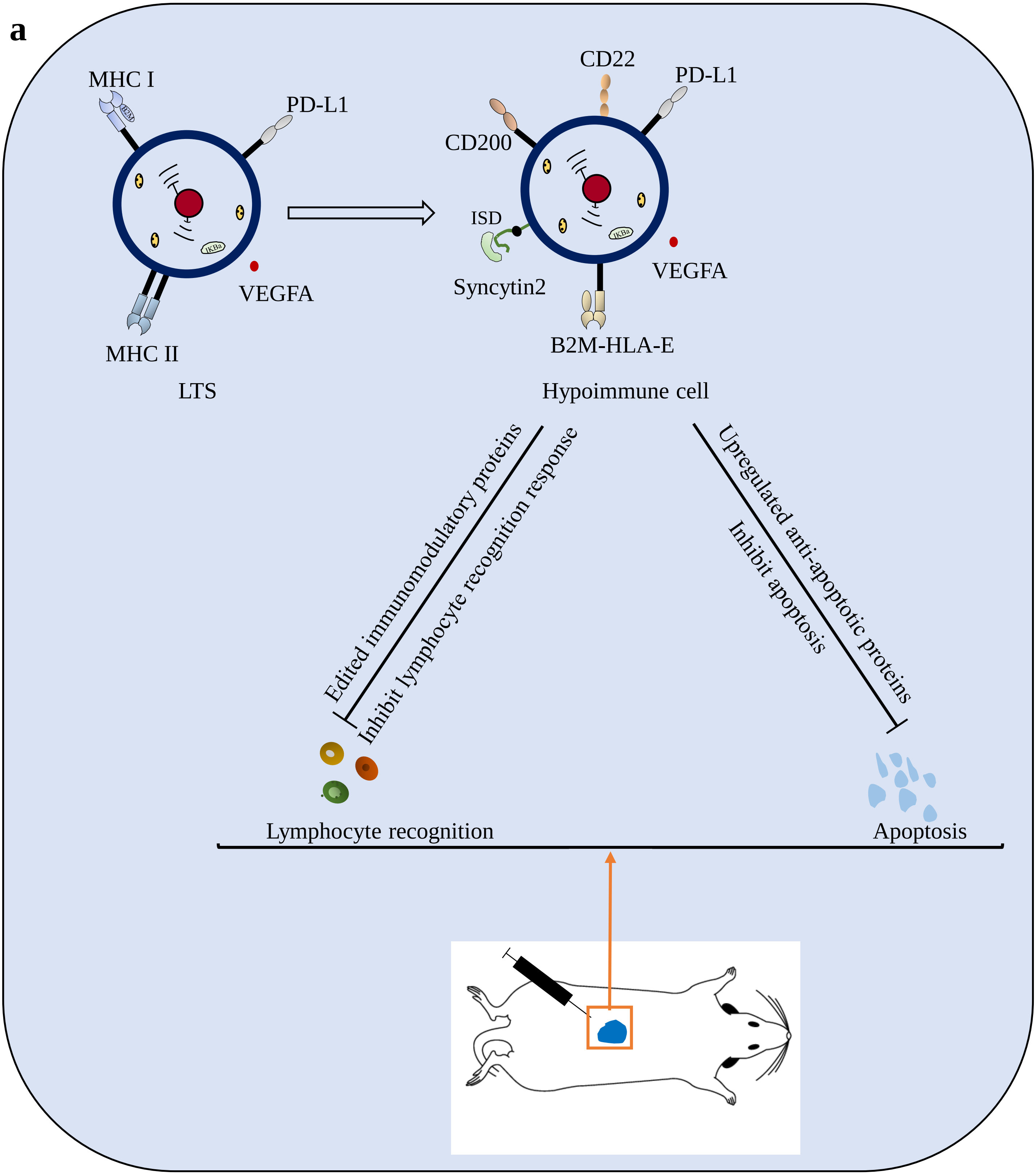

FigS5. Schematic diagram of hypoimmune muscle progenitor cells reducing allogeneic immune rejection

a. Cells face two challenges after transplantation: recognition and killing by lymphocytes, and cell apoptosis induced by the transplantation environment. Hypoimmune cells aim to address these two challenges through editing of immunomodulatory proteins and overexpression of anti-apoptotic proteins.
